# 山陰・夢みなと博覧会にみる泉眞也の方法論

**DOI:** 10.12688/f1000research.162338.1

**Published:** 2025-04-16

**Authors:** 江藤光紀 江藤

**Affiliations:** 1 Faculty of Humanities and Social Sciences, University of Tsukuba, Tsukuba, Ibaraki, 3058571, Japan

**Keywords:** International Exposition, Regional Exposition, Izumi Shinya, Japan Expo, Sakaiminato

## Abstract

泉眞也(1930-2022)は1970年の大阪万博から2005年の愛知万博まで日本で開催された全ての万博に重要なポジションで参加し、特に1990年の大阪・花と緑の博覧会、愛知万博では総合プロデューサーとして全体を統括した。

万博のようなメガイベントではプロデューサーたちがどのように博覧会を構想し実現へと導いたのかを評価することは簡単ではない。そこで本短信では1997年に鳥取県境港市で行われた山陰・夢みなと博覧会の構想を取り上げ、その策定プロセス、および独自性を要約した。入場者数で言うと愛知万博の10分の1程度の地方博だが、そこには泉とそのチームの博覧会実現の手法が端的に表れていると考えられる。

この博覧会は1996年の東京世界都市博(中止)を受け、2001年の北九州博覧祭を経て、愛知万博に至る泉の博覧会プロデュースの発展の一段階と意味づけることができよう。

## Abstract (en)

**Abstract:**

Izumi Shinya (1930-2022) participated in all the international expos held in Japan from the 1970 Osaka Expo to the 2005 Aichi Expo in important positions, and in particular oversaw the entire project as general producer for the 1990 Osaka International Garden and Greenery Exposition and the Aichi Expo.

It is not easy to evaluate how producers conceived and realised a mega-event such as an international exposition. In this short report, I have taken a look at the concept of the San'in Yumeminato Expo held in Sakaiminato City in 1997, summarising its formulation process and uniqueness. In terms of the number of visitors, it was a regional exposition with around one-tenth the size of the Aichi Expo, but it clearly represents the methods used by Izumi and his team in order to realise expositions.

This exposition can be seen as a step in the development of Izumi's exposition production, which followed the 1996 Tokyo World City Exposition (cancelled), through the 2001 Kitakyushu Expo (Kita Kyushu Hakuran Sai), and on to the Aichi Expo.

## はじめに

泉眞也 (1930-2022) は 1970 年の日本万国博覧会（大阪万博）から 2005 年日本国際博覧会（愛知万博）まで、日本で行われた全ての万博に関わり、またこの間に開催された多くの海外国際博にもプロデューサーやアドヴァイザーとして参加した。国際花と緑の博覧会（90 年大阪花博）と愛知万博では総合プロデューサーとして博覧会を統括するなど、この分野の第一人者だったと言ってよい。しかし泉がどのような思想を持ち、それを万博の現場にどう反映させてきたのかについてはこれまで顧みられることが少なかった。

　国際博覧会のようなメガイベントは政治や行政、企業の思惑など様々な要因を反映し、複雑なプロセスを経て成り立つため、プロデューサーのアイディアもそうしたものに規定・制限される。また万博に限らず博覧会は会期終了後には限られた恒久施設を除き解体されてしまう。種々の資料、公式記録や跡地から博覧会の構想の過程と成果をある程度追うことはできるが、万博という巨大なイベントに個々のプロデューサーの意図や思想を読み取ることはたやすくない。

そこでこの短信では、1997 年に鳥取で行われた山陰・夢みなと博覧会を対象に、どのようなプロセスを得て泉がチームを形成し理念や基本構想を博覧会に具現化したのかを明らかにする。この前年、1996 年に予定されながら直前になって中止に追い込まれた東京世界都市博にも泉は総合プロデューサーとして関与しており、筆者はすでにこの博覧会について論じたが（ 江藤光紀、2024年）、両者からは愛知万博に向けて段階的に発展する泉の方法論が浮かんでくる。

山陰・夢みなと博覧会は 1980 年代後半に地方博が粗製乱造されたという反省から通産省が設置したジャパン・エキスポ制度の認定を受けた7番目の博覧会で、泉は自らを支えるメンバーを要所に据え、総合プロデューサーとして構想から実現まで一貫して指揮を執った。コンパクトだが、それだけ一層彼の思想や方法論がダイレクトに表れている。

## 開催決定まで

夢みなと博覧会は境港市の開港 100 周年の記念事業として 1992 （平成 4）年に発案され、翌年 4 月 1 日に県商工労働部商工振興課に 2 名の専門職員が配置されることでスタートした（以下、博覧会の準備のプロセスについては山陰文化経営研究所 1998 年第一章「山陰・夢みなと博覧会の誕生」並びに『公式記録』年表、290-293 頁を参照）。同年秋、泉は後に本博覧会の構想を具体化していくことになるメンバーと共に鳥取県を広域にわたって視察した後、境港市長・黒見哲夫と面会し、同市で博覧会をジャパン・エキスポとして開催したいという要望を受け、11 月 17 日にアドヴァイザーに就任する。

当時博覧会は「税金のムダ遣い」というイメージが根強くあり、1983 年より知事を務めていた西尾邑次は当初必ずしも乗り気ではなかったようだが、泉チームは知事へのヒヤリングを通じ、3期目に入って独自色が出てきていた西尾県政の特徴をベースにコンセプトを作成した。

1994 年 3 月には最初の「基本構想（素案） 」が完成、地方が自立型生活圏を形成して地域産業を高度化し、地域による独自交流によって国際的な拠点を形成するという目標がいくつかのテーマにブレイクダウンされた。冷戦の終焉をきっかけにした韓国・中国・ロシアといった環日本海対岸諸国との連携、また境港の港湾施設・中国横断自動車道・米子空港といった交通網の整備をきっかけとする山陰・山陽・四国・関西の地域間の連携（ コミュニケーション・デザイニング研究所、1994 年、1 頁）、そして臨海公園を設置して全県公園化構想を推進し、鳥取県の魅力を内外に知らしめる（同、4 頁）という西尾県政の方向性は博覧会コンセプトへと的確に落とし込まれている。あわせて名称「山陰・夢みなと博覧会」、テーマ「翔け、交流新時代へ。 」、サブテーマ「環日本海交流の発展」「新たな海遊文化の創造」も設定された。

これを受けて設置された博覧会準備室は、6 月 1 日に泉に総合プロデュースを委嘱、同時にジャパン・エキスポへの認定申請を行った。ジャパン・エキスポは産業振興を主眼としていたため、国際交流を打ち出した同博に対し、所轄の通産省の当初の反応は必ずしも良くはなかった。また環日本海交流というコンセプトも新潟のイメージが強かったようだ（ 山陰経済経営研究所、1998 年、17-18 頁）。しかしジャパン・エキスポは地方通産局が本省に申請する形になっており、担当だった中国通産局長の強い後押しで、同年 9 月に認定が下りた。

## 会場計画

会場計画案は最初に作られた「基本構想（素案） 」の中にすでに提示されており、細部の異同はあるものの大枠のアイディアは最終案まで踏襲された。通常会場計画は基本構想がある程度固まってから策定されるが、泉はプランに具体性を持たせるため最初に絵を描くという信条を持っていた。作成プロセスは素案に段階的に提示されており、泉の博覧会の設計方法や理念を知る上で極めて興味深い。

まず境港の立地特性を中国・北朝鮮・ロシアの国境を通り日本海に注ぐ豆満江と鳥取を結んだ環日本海交流軸、そして鳥取を中心に日本海に沿って伸ばした日本海国土軸の交点と捉える。次に中国横断自動車道を、太平洋・瀬戸内海・日本海という三つの海の間にある四国横断軸・山陽国土軸・中国往還軸を結び付ける道路と捉え、山陰軸の中心に位置する境港を、その交流の窓口と位置付ける（ コミュニケーション・デザイニング研究所、1994 年、16 頁）。こうした大きな視点からのとらえ方は、博覧会でどの地域と交流するかの基礎アイディアとなる。

景観特性を踏まえた会場計画案は、二つの基本軸から構成される。弓ヶ浜半島の両側、西の中海、東の美保湾を結んだ軸は延長すると古都・長安に至り、また美保湾から上った太陽が中海に沈むラインでもあることから「大陸・太陽軸（交流の道） 」と名付けられた。また大山から北西に伸び島根半島に向かうラインは、軸の両端に山々を臨み、東西を海に挟まれていることから、「海幸山幸軸（創造の道） 」と名付けられた（同、17 頁）。

この二つの軸を会場のゾーニングへと反映することで配置に物語性が生まれる。軸の交点、つまり会場の中心となる「夢みなとテーマゾーン」は環日本海交流を体験し、鳥取の可能性を理解する場である。「大陸・太陽軸」上には中国・韓国・ロシアといった対岸諸国との理解と交流を深める「環日本海交流ゾーン」、鳥取県の産業、文化、歴史、未来へのビジョンを知るための「山陰・鳥取発見ゾーン」が置かれる。 「海幸山幸軸」上には海水浴場、遊園地、オートキャンプ場などのレジャー施設を複合化した「海遊文化ゾーン」、この地域で発達した氷温技術を生かした食を楽しむ「海幸山幸ゾーン」が配置され、それぞれのゾーニングの意味合いにそったパビリオンや施設へと展開されるのである（同、18-20 頁）。

## プロデューサー体制・ディレクター制度

この博覧会は実施体制においてもユニークだった。泉は事務所や会社組織を持たず、緩やかにつながる人脈の中から必要に応じて人材を適材適所に配置した。夢みなと博覧会では当初より泉にとりわけ近しいメンバーが加わっており、準備が進む中で正式なポジションに就任していく。

1995 年 4 月 1 日にはコミュニケーション・デザイニング研究所の代表・福井昌平が事業・企画のサブ・プロデューサー、株式会社 SD の代表・古見修一が会場・演出のサブ・プロデューサーに委嘱される。福井はコーポレート・アイデンティティの草分けとして 80 年代より泉フォーラムに参加し泉と親交を持っており、都市博をきっかけに博覧会事業も手掛けるようになっていた。古見はつくば科学博をはじめ、泉をサポートしながら多くの博覧会事業を手掛けていたベテランだ。

こうした体制のもと、さらに 2 人のチーフディレクター、7 人の専門ディレクターを置いたのも、この博覧会の特徴である。ディレクターには若手が選任され、博覧会協会の職員がより気軽に相談できる形が整えられた。チーフディレクターには SD の沢田裕二とコミュニケーション・デザイニング研究所の石川勝が就任したが、彼らはいずれも古見、福井の下にいたスタッフだ。ディレクター陣が設置されたのは実施計画の策定後の 95 年 9 月のことだが、2 人はそれ以前から（特に石川は最初の鳥取視察から）博覧会の準備に関わっており、就任後は展示演出ディレクター（沢田）、広報ディレクター（石川）も兼任しつつ、協会スタッフの立場にたったアドヴァイスを行った。博覧会事業では各段階で携わるメンバーが入れ替わることが普通であり、企画・構想を練ったスタッフが最終実施まで同一のポジションで、責任を持ってやり遂げるスタイルは当時まだ異例であった。

さらにプロデューサーチームの立てた企画や立案を協会が聞くプロデューサー会議は、途中から大量の業務案件をプロデューサーの助言のもと、多くの部署が集まって一気に解決する業務調整会議に名称変更され、協会スタッフがより積極的に業務に携わる姿勢を生んだ（ 山陰経済経営研究所、1998 年、35-36 頁）。

泉チームの博覧会構想と基本理念は、こうした運営体制によって実現されていったのである。

## 成果

　博覧会の理念を実装するために、最終会場計画案では中心部を「夢ロード」で囲い、この内部に協会企画事業として「環日本海交流村」「産業未来館」「地域交流館」「ふるさとパーク」を配置することとなった （ 山陰・夢みなと博覧会協会、1997 年、 22-23 頁）。これらは協会が単独事業として全てを自前で作るのではなく、関係の深い県の各部署や各種団体が積極的に参加することで大きなにぎわいを見せた。

「環日本海交流村」にはロシア沿海地方、中国吉林省・大連市、韓国江原道、モンゴル中央県が出展し、歴史や文化を紹介したほか、国際色豊かなイベントを開催した（鳥取県の友好都市・河北省は単独パビリオンを出展）。地方博でこのような大掛かりな国際交流がなされたのは画期的であった。また地域交流館には、大阪府、兵庫県、島根県、岡山県、広島県、山口県、香川県、徳島県、高知県に鳥取を加えた 1 府 9 県が参加した。来場者は展示などで理解を深めた後、未来の物語をおとぎ話風に仕立てたミュージカルを楽しんだ。

またこの博覧会ではボランティアセンターが組織されたが、協会の傘下に入らず自主的に活動内容を考え、現場の改善に活かした点にも大きな意義があった。ボランティアも予想の三倍に当たるのべ 15000 名近くが参加した（ 山陰経済経営研究所、1998 年、53 頁）。

このほかにも泉チームと博覧会協会は詳細な動員収支予測調査を数度にわたって行い、黎明期にあったインターネットも活用した広報活動を行うことで、192 万人の来場者を記録した。ジャパン・エキスポにおいては多いわけではないが、人口 60 万人の県をにぎわせるには十分な数字であった。

## 山陰・夢みなと博覧会の意義

本稿では日本の万博史における泉の業績を振り返るという視点から、山陰・夢みなと博覧会の成り立ちと成果を追ったが、最後にその意義をまとめたい。

本博覧会を立ち上げた段階で泉は東京世界都市博に注力していたが、95 年 5 月 31 日に青島東京都知事が中止を表明したことにより都市博は死産に終わった。夢みなと博覧会はそれに比べればずっと小規模だが、両者を比較すると興味深いことが分かる。都市博は国際博としての規模を保ちながらも、国単位の万国博から都市単位の博覧会へと組み替えが図られた。一方、夢みなと博が目指したのは環日本海、山陰・山陽・四国・関西といった地域間交流である。

そもそも地域間交流は西尾知事の政治理念だったが、それが泉チームの知見を通じて博覧会へと具現化されたと言えよう。当初博覧会事業に乗り気でなかった西尾も、だからこそトップとして決断を下し、各所の理解・協力を取り付け、積極的に出展招致に動いた。泉が提案した「夢みなとタワー」も、西尾の英断で実現したという。泉の母親の実家が境港で回漕店を営んでいたという過去、またチーフプロデューサーの福井が鳥取出身だったという縁も、泉チームと行政の間の信頼関係を強めたようである（ 山陰経済経営研究所、1998 年、4 頁）。

総合プロデューサー体制、ボランティアの積極的な起用などこの博覧会から後に引き継がれた成果の他にも、「環日本海交流村」における展示・物販・飲食・催事の一体化なども重要な成果として挙げられる。テーマパークでは一般化していたこの手法を、泉は都市博の「オイコスパーク」で博覧会に導入しようとし、夢みなと博で実現をみた。また夜間の一大スペクタクルとして話題を呼んだ「スペーシア」はフランスの演出家イヴ・ペパンによるものだったが、当初ぺパンは都市博に参加することになっていた。

この博覧会の成功により、泉は北九州市が計画していたジャパン・エキスポの総合プロデュースを依頼された。夢みなと博、北九州博覧祭といった地方博の経験は愛・地球博にも活かされていったと思われる。

## Data Availability

本論文には関連データがない。文献には未刊行の資料が含まれており、これについては所蔵先を参照のこと。同文献は鳥取県や博覧会協会に公式に提出された、外部からの要望に応じて公開することを前提とした鳥取県と博覧会協会の公式資料である。

## References

[ref1] 江藤 光紀 : 未完の万都市博 － 東京世界都市博における泉眞也の構想をめぐって. *」、暮沢剛巳他『万国博覧会と日本 － アートとメディアの視点から』* (勁草書房、2024).

[ref2] 山陰経済経営研究所: *夢みなと博の贈り物 － 博覧会による地域活性化事例の研究.* (山陰経済経営研究所、1998).

[ref3] 山陰・夢みなと博覧会協会: 山陰・夢みなと博覧会公式ガイドブック. 1997.

[ref4] 山陰・夢みなと博覧会協会: 山陰・夢みなと博覧会公式記録. 1998.

[ref5] コミュニケーション・デザイニング研究所: 山陰・夢みなと博覧会 基本構想(素案). *1.本編 平成 6 年 3 月 (内部報告書、未刊行、福井昌平氏所蔵).* 1994.

