# Supplementary material for: Izumi Shinya's methodology at the San'in Yumeminato Expo
Source: F1000Res. 2025 Apr 16;14:441. [Article in Japanese] [Version 1] doi: 10.12688/f1000research.162338.1 (PMC12090981; doi:10.12688/f1000research.162338.1)
Supplement: Peer review history

# Reviewer report: Reviewer response for version 1

**DOI:** 10.5256/f1000research.178525.r378302

**Published:** 2025-06-10

**Authors:** Kenji Saotome

**Affiliations:** 1 Osaka International University, Osaka, Japan

**■** **概要**

 本論文は、日本における博覧会プロデュースの第一人者である泉眞也氏（1930–2022）の方法論を、1997年に鳥取県境港市で開催された山陰・夢みなと博覧会を通じて考察するものである。筆者は、本博覧会を1996年の東京世界都市博（開催中止）および2005年の愛知万博との連続性の中に位置付け、泉の博覧会思想とチーム編成、空間構成や運営体制の特徴を丁寧に描いている。

**■** **総合評価**

 本稿は、泉眞也氏という個人の思想と実践を掘り下げ、従来あまり注目されてこなかった地方博覧会の実態に光を当てた意欲的なケーススタディである。特に、未刊行の一次資料や関係者への聞き取りを適切に用いており、資料的価値が高い。社会科学・人文科学分野における博覧会研究、イベント研究、地域政策研究、文化経営研究に対する貢献度は大きいと評価できる。

**■** **科学的妥当性と改善提案**

**構成・明瞭性**

 章立てが明確で、論旨の展開も分かりやすい。日本語としての表現も丁寧である。ただし、一部の段落では冗長な説明が見られるため、簡潔化により論旨の焦点がさらに明確になる。

**方法と再現性**

 歴史的事例分析として方法論に問題はないが、使用資料のアクセス性（特に未刊行資料）については、より明確な記述が望まれる。たとえば、資料名に加えて所蔵機関・閲覧条件などを補記してほしい。

**データ可用性**

 基本的に文章での記述によって構成されており、図表や地図が含まれていない。文中で述べられる「軸」の構成やゾーニングの視覚的補助があると、読者の理解をより深められる。図表・略図の追加を推奨する。

**学術的貢献**

 国内外の博覧会研究の中で、プロデューサーに焦点を当てた論考は少なく、学術的独自性が高い。また、地域政策との接点を指摘する点も有意義である。ただし、博覧会研究、イベント研究における既存文献との比較検討がもう少し欲しい。国際的な事例との対照にも触れると、さらなる意義が見えてくる。

**参考文献・情報源**

 信頼できる公的資料と一次資料を活用しており、高く評価できる。非公開資料については、その性質とアクセス条件の補足があれば、透明性がより確保される。

**■** **推奨される修正点**

**図版の追加**

 空間構成（「大陸・太陽軸」「海幸山幸軸」）などを視覚的に理解できるよう、簡易な図版または会場マップを挿入してください。

**資料の入手可能性に関する補記**

 未刊行の資料については、所蔵先（例：福井氏所蔵、鳥取県庁など）や、閲覧・引用の条件についての説明を加えてください。

**理論的背景や比較の強化**

 泉の方法論をより普遍的な「イベント・マネジメント理論」や「市民参加型ガバナンス」などの視点から位置づけると、国際的読者にとっての意義が明確になります。特に愛知万博などとの手法的比較があると説得力が増します。

**■** **結論・最終判断**

 本稿は、歴史的・実践的に貴重な知見を提供しており、査読付きで公開されるに値する内容を備えている。上記の点について軽微～中程度の修正を加えることで、より多くの読者にとって有益な論文となるであろう。

**■** **承認ステータス**

 条件付き承認（Approved with Reservations）

（該当する場合は要回答）統計分析および解釈は適切ですか。

対象外（統計を使っていない）

本研究は明確かつ正確に提示されたものであり、最新の文献を引用していますか。

はい

方法と分析について第三者による再現が可能となるよう十分な詳細が提示されていますか。

はい

結果の基礎となるソースデータはすべて入手可能で再現性を十全に保証していますか。

ソースデータは不要

結論は結果により妥当な裏付けを得ていますか。

はい

研究設計は適切で学術的価値がありますか。

はい

Reviewer Expertise:

博物館学、万博史、近現代史

I confirm that I have read this submission and believe that I have an appropriate level of expertise to confirm that it is of an acceptable scientific standard, however I have significant reservations, as outlined above.

# Reviewer report: Reviewer response for version 1

**DOI:** 10.5256/f1000research.178525.r379503

**Published:** 2025-05-19

**Authors:** Takayuki Yoshida

**Affiliations:** 1 Osaka Metropolitan University, Osaka, Japan

　日本で開催されてきた万国博覧会、都市博に参加してきた泉眞也、なかでも山陰・夢みなと博覧会に焦点をあて、泉の策定プロセス、独自性を要約したものであると理解した。すなわち、その独自性とは、会場計画を基本構想が固まる前から策定したこと、地域の政治課題を拾いつつマクロな視点で、環日本海交流、地域間交流という2軸の明確化かつ大きなビジョンを示したこと、泉の人脈で構想から実施に至るまで同一スタッフが担ったこと、多くの部署が集まって一気に解決する業務調整会議により、スタッフが積極的に関わる姿勢を生んだことなどである。

 　成果としては、ビジョンに照らした交流を生み出し、ボランティア活動の自主性などにも意義があったという。展示・物販・飲食・催事の一体化なども重要な成果だと指摘があった。

 　大阪万博開催中であり、成功の有無、意義が問われる中、日本の博覧会の歴史に遡り、博覧会の意義を検証しようとする本稿は、時宜にかなった社会的意義の高い短信である。過去にさかのぼり、小規模展覧会に着目する研究方法は、非常に好感がもてる。それに対して、「はじめに」で、先行研究として、「筆者はすでにこの博覧会について論じたが（ 江藤光紀、2024年）」とあるが、本研究の学術的意義をより明確にするために、先行研究の内容と本稿の結論の異同について、「はじめに」や「結論」で、明瞭に言及していただきたい。こうした点から、「研究設計は適切で学術的価値がありますか」について一部該当とした。

 　また、本稿の問い（研究の目的）と結論の関係が、やや不明確な点があることから、「結論は結果により妥当な裏付けを得ていますか。」について一部該当とした。

 　問いは、「どのようなプロセスを経て泉がチームを形成し理念や基本構想を具現化したのか明らかにする」とあり、策定プロセスのみに争点を当てるかのようである。一方で、要旨では、「その策定プロセス、独自性を要約した」とある。本短信の目的が、策定プロセスだけでなく、その独自性を明らかにする点であるならば、本文中の問いでも明記されたい。

 　加えて、「山陰・夢みなと博覧会の意義」が本短信の結論にあたるようにも思える。しかし、上記問いに対する結論のまとめがなく、読者からするとついていけない嫌いがある。「総合プロデューサー体制」が結論の1つと考えられるが、その具体的ななかみ、もしくは、前頁の記載のいずれの箇所をさすのか明記していただきたい。ボランティアの積極的な起用なども意義として挙げられているが、成果で軽く言及されているにすぎない。本短信の目的が独自性を明らかにするということであれば、結論としては、策定プロセスだけでなく、成果にも独自性が認められるはずである。もちろん、策定プロセスのみでも構わないが、その点の整理も不十分と見受けられた。

 　謝辞に「福井昌平氏（CI 戦略プロデューサー、イベント学会副会長）からは当時のお話しを聴かせていただいた他、多くの資料をご提供いただきました。」とあるが、論文の再現可能性からすると、下記文章についてインタビューなどの根拠資料の明示がもとめられよう。それゆえ、「方法と分析について第三者による再現が可能となるよう十分な詳細が提示されていますか。」について一部該当とした。

 ・「開催決定まで」の末文「担当だった中国通産局長の強い後押しで、同年 9 月に認定が下りた。」

 ・「会場計画」の本文2行目～「泉はプランに具体性を持たせるため最初に絵を描くという信条を持っていた。」

 ・「プロデューサー体制・ディレクター制度」の3段落目「博覧会協会の職員がより気軽に相談できる形が整えられた。」

 ・「プロデューサー体制・ディレクター制度」3段落目末文「博覧会事業では各段階で携わるメンバーが入れ替わることが普通であり、企画・構想を練ったスタッフが最終実施まで同一のポジションで、責任を持ってやり遂げるスタイルは当時まだ異例であった。」

（該当する場合は要回答）統計分析および解釈は適切ですか。

対象外（統計を使っていない）

本研究は明確かつ正確に提示されたものであり、最新の文献を引用していますか。

はい

方法と分析について第三者による再現が可能となるよう十分な詳細が提示されていますか。

一部該当

結果の基礎となるソースデータはすべて入手可能で再現性を十全に保証していますか。

ソースデータは不要

結論は結果により妥当な裏付けを得ていますか。

一部該当

研究設計は適切で学術的価値がありますか。

一部該当

Reviewer Expertise:

文化政策

I confirm that I have read this submission and believe that I have an appropriate level of expertise to confirm that it is of an acceptable scientific standard, however I have significant reservations, as outlined above.

# Reviewer report: Reviewer response for version 1

**DOI:** 10.5256/f1000research.178525.r378296

**Published:** 2025-05-02

**Authors:** Nobumichi Ariga

**Affiliations:** 1 Hitotsubashi University, Tokyo, Japan

本稿は1997年の山陰・夢みなと博覧会（以下、「夢みなと博」とします）を題材に、その総合プロデューサーを務めた泉眞也の仕事を論じた短報です。日本の博覧会史における泉の重要性からして、その思想や方法論を明らかにしようとする本研究には学術的意義があるでしょう。その際、「はじめに」で述べられているように巨大なイベントからプロデューサーの貢献を読み取ることは一般に容易でないため、あえて小規模な博覧会に注目するという研究アプローチは支持できます。また、すぐあとで述べることを除き、論考の構成も短報としてはおおむね適切と考えます。

 本稿の問題点と考えられるのは、まず、結論が明確に述べられていないことです。泉の「思想」と「方法論」が本稿の主題である旨が「はじめに」で書かれていますが、本稿の最後は夢みなと博の意義についての記述で終わっており、同博覧会に見られる泉の「思想」と「方法論」についてまとめられていません。本文中、「会場計画」と「プロデューサー体制・ディレクター制度」の二つの節で書かれていることがそれらに相当するように思われましたが、「おわりに」の節を設けるなどしてあらためて言語化し、議論をまとめていただく必要があると思います。またこの意味での結論は、要旨にも含めて記すべきではないかと考えます。

 次に、先行研究についてです。著者はこれまでに泉の仕事について複数の文章を発表されていると思いますが、本稿ではそのうち東京世界都市博を論じたものだけが参照されています。関連するものを先行研究としてすべて挙げておくほうが、読者にとっても有益ではないかと思います。本論にあたる各節では、ところどころ、説明を補足したり、出典の注記を加えたりしていただきたい箇所が見られます。順番に以下に記します。

- 「開催決定まで」の第1段落で参照されている資料の著者は、「山陰文化経営研究所」でなく「山陰経済経営研究所」ではないでしょうか。
- 「会場計画」の第1段落、「泉はプランに具体性を持たせるため最初に絵を描くという信条を持っていた」という記述の根拠は何でしょうか。
- その次の二つの段落では、境港の立地特性とそれを踏まえての会場計画案が述べられていますが、図がないと非常にイメージしづらいです。説明のための図版を加えてほしいと思います。資料に含まれている実際の図を載せるのでなくても、説明用に模式図を描いていただくのでも構いません。
- 「プロデューサー体制・ディレクター制度」の第2段落と第3段落にも出典の注記が必要と思います。特に第3段落の最後、「企画・構想を練ったスタッフが最終実施まで同一のポジションで、責任を持ってやり遂げるスタイルは当時まだ異例であった」というのは、夢みなと博の資料を見ただけでは分からないことではないでしょうか。
- 「山陰・夢みなと博覧会の意義」の第4段落で書かれている内容についても文献参照を要します。この参考文献は東京都市博に関する著者の論考になるかと思います。

以上

（該当する場合は要回答）統計分析および解釈は適切ですか。

対象外（統計を使っていない）

本研究は明確かつ正確に提示されたものであり、最新の文献を引用していますか。

一部該当

方法と分析について第三者による再現が可能となるよう十分な詳細が提示されていますか。

はい

結果の基礎となるソースデータはすべて入手可能で再現性を十全に保証していますか。

ソースデータは不要

結論は結果により妥当な裏付けを得ていますか。

一部該当

研究設計は適切で学術的価値がありますか。

はい

Reviewer Expertise:

科学技術史、博覧会史

I confirm that I have read this submission and believe that I have an appropriate level of expertise to confirm that it is of an acceptable scientific standard, however I have significant reservations, as outlined above.

# Reviewer report: Reviewer response for version 1

**DOI:** 10.5256/f1000research.178525.r378299

**Published:** 2025-04-30

**Authors:** Kazuhiro Nose

**Affiliations:** 1 Ritsumeikan University, Kyoto, Japan

　本論文は05年の愛知万博総合プロデューサーとして知られる泉眞也の功績を、1997年山陰・夢みなと博覧会における彼の構想から明らかにしようとしたものである。本論文の独創性は、山陰・夢みなと博覧会という、これまで研究者がかならずしも重要視してこなかった博覧会に焦点を当てることで、泉眞也という「万博人」の個性の一端を解き明かした点にある。とくに同博覧会で泉が環日本海世界のなかに鳥取を位置付ける構想を実現させたという事実は、以後の博覧会コンセプトとの異同を浮かび上がらせるという点で興味深い。

 　日本における万博をめぐる叙述は、1970年大阪万博と2025年大阪・関西万博を直線的に結びつける傾向がある。しかし、その間には数多くの地方博、そして愛知万博が取り行われていたことを無視すべきではない。そして、そうした「忘れられた」万博の中心にいた人物のひとりが泉であり、彼の博覧会にかける思想・方法論を明らかにすることは、典型的なメガ・イベントに集約されえない、別の仕方での万博がありえた可能性に光を当てることにつながる。短報という特性上、本論文はこの山陰・夢みなと博覧会の経験がのちにどのように生かされたのか、あるいは生かされなかったのかまで論じているわけではない。また、博覧会の成果を評価するうえで不可欠といえる、来場者の反応も来場者数以外に検討されていない。しかし、それらは形式的な制約ゆえのことであり、本論文の価値を低めるものではない。本論文は、従来の万博史叙述を大きく塗り替えるような研究の一ピースとして、高い学術的価値を秘めている。

（該当する場合は要回答）統計分析および解釈は適切ですか。

対象外（統計を使っていない）

本研究は明確かつ正確に提示されたものであり、最新の文献を引用していますか。

はい

方法と分析について第三者による再現が可能となるよう十分な詳細が提示されていますか。

はい

結果の基礎となるソースデータはすべて入手可能で再現性を十全に保証していますか。

ソースデータは不要

結論は結果により妥当な裏付けを得ていますか。

はい

研究設計は適切で学術的価値がありますか。

はい

Reviewer Expertise:

歴史学・国際関係史

I confirm that I have read this submission and believe that I have an appropriate level of expertise to confirm that it is of an acceptable scientific standard.
